# Cereal-Based Gluten-Free Food: How to Reconcile Nutritional and Technological Properties of Wheat Proteins with Safety for Celiac Disease Patients

**DOI:** 10.3390/nu6020575

**Published:** 2014-01-29

**Authors:** Carmela Lamacchia, Alessandra Camarca, Stefania Picascia, Aldo Di Luccia, Carmen Gianfrani

**Affiliations:** 1 Department of Food Science, University of Foggia, Via Napoli 25, 71100 - Foggia, Italy; E-Mails: c.lamacchia@unifg.it (C.L.); a.diluccia@unifg.it (A.D.L.); 2 Institute of Food Sciences-CNR, Carmen Gianfrani, ISA-CNR, Via Roma 64, 83100 - Avellino, Italy; E-Mails: acamarca@isa.cnr.it (A.C.); s.picascia@isa.cnr.it (S.P.); 3 Institute of Protein Biochemistry-CNR, Via P. Castellino, 111, 80131 – Naples, Italy

**Keywords:** Celiac Disease, cereals, wheat gluten, dough, immune toxicity

## Abstract

The gluten-free diet is, to date, the only efficacious treatment for patients with Celiac Disease. In recent years, the impressive rise of Celiac Disease incidence, dramatically prompted changes in the dietary habit of an increasingly large population, with a rise in demand of gluten-free products. The formulation of gluten-free bakery products presents a formidable challenge to cereal technologists. As wheat gluten contributes to the formation of a strong protein network, that confers visco-elasticity to the dough and allows the wheat flour to be processed into a wide range of products, the preparation of cereal-based gluten-free products is a somehow difficult process. This review focuses on nutritional and technological quality of products made with gluten-free cereals available on the market. The possibility of using flour from naturally low toxic ancient wheat species or detoxified wheat for the diet of celiacs is also discussed.

## 1. Introduction

In the past 100 years, the human being has experienced a biological transformation with a speed greater than the trend of the last millennia. Over the past seventy years, the diet of Western man has been totally revolutionized: farm food, once characterized for being fresh and free of toxin residues, additives and preservatives, has been almost completely replaced by food products of an industrial chain that is, in the great majority of cases, oriented towards profit and the creation of induced needs. All this has radically changed, and still continues to change, the biological systems in humans leading to metabolic syndrome, allergies and intolerances, among both children and adults, in developed and developing countries.

In this context, in the last two decades, a series of epidemiologic studies have shown a particular increase in Celiac Disease (CD), a life-long intolerance to gluten proteins (the seed storage proteins) present in most cereals [1], both in the United States and Europe, and in developing countries [2,3]. In these subjects, the consumption of cereals containing gluten causes a chronic inflammatory process leading to lesions in the small intestine and a dysfunction in nutrient absorption [1]. The CD treatment, therefore, is based on a strict gluten-free diet throughout the patient’s lifetime. Though this dietary regimen guarantees the full recovery of small intestine architecture and functions, for many patients it is strongly restrictive, especially for social events and during travelling. In addition, it is a great task for the food industry to provide safe food that partly resembles in taste and appearance pasta, bread and other baked goods. Furthermore, the nutritional importance of wheat proteins should not be underestimated, particularly in less developed countries where bread, noodles and other products (e.g., bulgur, couscous) may represent substantial diet components.

## 2. Celiac Disease

Celiac disease is the most common food induced enteropathy in humans. CD is strongly associated with particular HLA genotypes, as only individuals carrying the DQA1*0501 and DQB1*0201 (DQ2), or DQA1*0301 and DQB1*0302 (DQ8) alleles develop the disease. Gluten intolerance presents a large variety of symptoms including gastrointestinal and extra-intestinal manifestations, though in some patients, particularly of pediatric age, the disease is completely symptomless [4,5]. Typical clinical manifestations of CD include chronic diarrhea, weight loss and anemia, mainly caused by malabsorption, as a direct consequence of intestinal villous atrophy. However, a significant proportion of patients have “atypical” form, characterized by extra-digestive symptoms, including skin lesions, isolated hypertransaminasemia, bone pains and fractures, and infertility [6]. Several serological screening detecting the CD-associated anti-tissue transglutaminase (or anti-endomysium) IgA antibodies, and followed by endoscopy, have shown that approximately 1% of the general population have celiac disease [7,8]. However, Maki and co-workers found that the total prevalence of CD is even higher, reaching 1.99% in the Finnish population [9]. The wide spectrum of clinical manifestations, together with the absence of clear symptoms in some cases, could explain why the prevalence of CD is so underestimated. Regarding the pathogenic mechanisms, it is widely accepted that CD is an immune mediated disorder, in which intestinal CD4+ T cells, highly reactive to dietary gluten, have a pivotal role in disease pathogenesis [10]. In addition, recent studies have indicated the prominent role of both innate immune cells and adaptive CD8+ T cells in damaging the mucosal tissue [10,11]. To date, the only treatment for celiac disease patients is the lifelong complete exclusion of the gluten from the diet. With such a dietary restriction, clinical symptoms, serological markers, and duodenal mucosa histology normalize [12], however gluten reactive T cells remain in the small intestine of patients on gluten-free diet, and re-exposure to the gluten results in immune activation and in mucosal damage. As a consequence, gluten avoidance is strongly recommended; nevertheless, dietary compliance is imperfect in a large number of patients for several reasons, including the need of flavor and palatability of gluten-free cereal-based food [13].

## 3. Gluten, the Dough “Treasure”

Gluten is one of the earliest protein fractions described by chemists, (a first description by Beccari was in 1728), and it is defined as the “cohesive, visco-elastic proteinaceous material” that remains when wheat dough is washed to remove starch granules and water soluble constituents [14,15]. Gluten is formed by storage proteins necessary for plant germination [16]. In these respects, the wheat storage proteins may not differ much from those of other cereals [14,17], however, the distinctive feature that makes wheat unique is, precisely, the visco-elasticity of gluten. When the grain is milled and mixed with water, storage proteins form a dough, capable of retaining gas bubbles. These properties make wheat suitable for the preparation of a great diversity of food products, including breads, noodles, pasta, and cookies, and it has been the subject of extensive attention by the food industry. Of note, in order to meet the consumer needs, food industries have made in the last fifty years an indiscriminate use of wheat gluten even in food that is naturally devoid, as sauces, chips, cold cuts.

**Figure 1:**
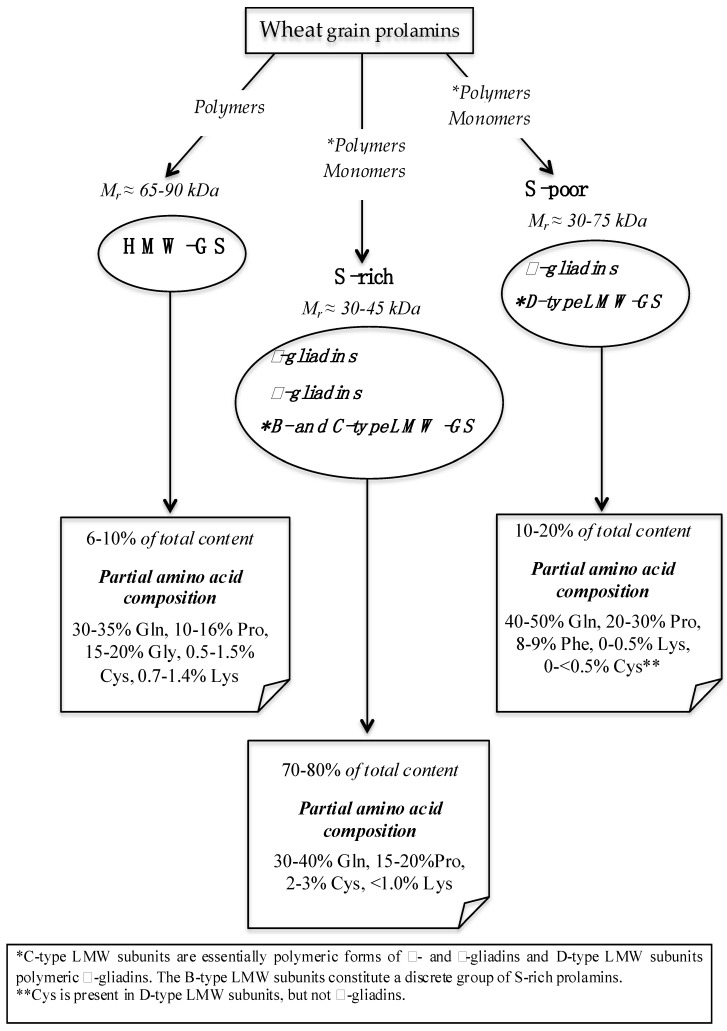
Schematic representation of types and peculiarities of wheat gluten proteins (gliadins and glutenins-GS; modified from [17]).

**Figure 2:**
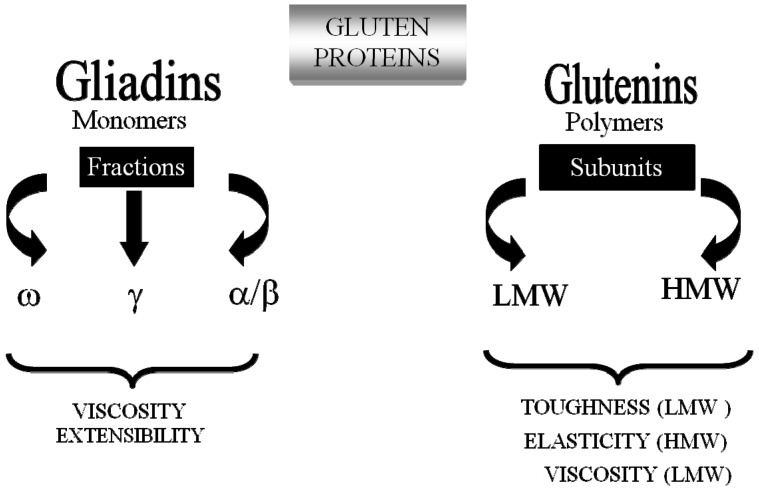
Gluten proteins: fractions and technological properties [23].

Gluten contains hundreds of proteins, which are present either as monomers or as oligo- and polymers, linked by inter-chain disulphide bonds [15,17], and characterized by high contents of glutamine and proline (namely prolamins), and by low contents of charged amino acids. The new system of classification, based on the availability of complete amino acid sequences, divided the gluten proteins in three broad groups: sulphur-rich (S-rich), sulphur-poor (S-poor), and high molecular weight (HMW) prolamins ([18], Figure 1). Traditionally, gluten proteins have been divided according to their solubility in alcohol-water solutions (e.g., 60% ethanol), as the soluble gliadins and the insoluble glutenins [19]. These properties are largely determined by the inter-chain disulphide bonds, with the glutenins consisting of disulphide-stabilized polymers. Reduction of these inter-chain bonds allows the separation of the glutenin subunits into low molecular weight (LMW) and high molecular weight (HMW) groups (Figure 2). In contrast, the alcohol-soluble gliadin fraction consists mainly of monomeric proteins, which either lack cysteine (ω-gliadins), or have only intra-chain disulphide bonds (α-type and γ-type gliadins) (Figure 2). However, small amounts of polymers related to the glutenins are also present in the gliadin fraction. These appear to differ from the alcohol-insoluble glutenins, as they have lower *M*_r_s and higher contents of LMW subunits [20,21,22].

**Figure 3:**
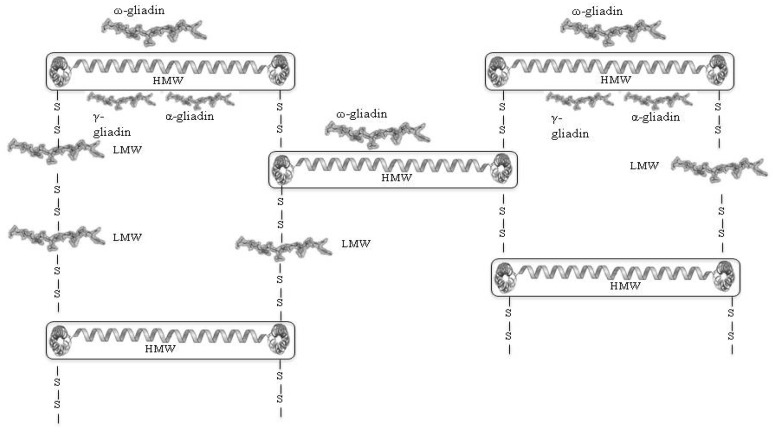
A structural model for wheat gluten in which the HMW subunits provide a disulphide-bonded backbone that interacts with other gluten proteins by disulphide bonds (LMW subunits) and non-covalent interactions (gliadins) (modified from [26]).

Both fractions are important contributors to the rheological properties of dough (Figure 2), though their functions are divergent. Hydrated gliadins have little elasticity and are less cohesive than glutenins, and contribute mainly to the viscosity and extensibility of the dough. In contrast, hydrated glutenins are both cohesive and elastic and are responsible for dough strength and elasticity [24,25]. A proper mixture of both fractions is essential for the quality of the end product. However, of particular importance are the glutenin polymers, and it is well established that strong (*i.e.*, highly visco-elastic) doughs contain high proportions of HMW glutenin polymers [24]. Numerous studies are consistent with the hypothesis that the HMW subunits form an elastomeric polymer network which provides a “backbone” for interactions with other glutenin subunits and with gliadins (Figure 3) [26,27]. There is no doubt that this network is mainly stabilized by inter-chain disulphide bonds [28,29]. Additional covalent bonds formed during dough making are tyrosine-tyrosine and thiyl-tyrosine crosslinks between gluten proteins [30,31,32]. However, the covalent structure of the gluten network is superimposed by non-covalent bonds (hydrogen bonds, ionic bonds, hydrophobic bonds) [33].

## 4. Formulation and Nutritional Value of Gluten-free Products

The food not allowed in the gluten free diet include: (a) all types of bread and food prepared with wheat flour, including kamut and spelt, rye, barley, triticale, or with ingredients from these flours; (b) food that contains wheat, or derivatives of gluten used as thickeners, such as hot dogs, salad dressing, sauces, canned, some types of cheese and cold cuts; (c) medicinal products that use gluten as binder in the pills or tablets.

Though from a nutritional point of view, gluten exclusion does not entail particular problems, being a mixture of proteins with low nutritional and biological value, the gluten-free diet creates enormous limitations, above all in the social activities related to food. In addition, this dietary therapy has, often low content of vitamins and ions, such as vitamins B and D, calcium, iron, zinc, and magnesium, as well as fiber [34,35,36]. Furthermore, one of the major risks is the risk to develop obesity and diseases related to metabolic syndrome [37]. However, the high technological value renders gluten almost indispensable in baked products, and its replacement, as structure-building protein, presents a major technological challenge for the food industry. Although many advances have been made in the preparation processes of gluten-free products, using starches, hydrocolloids, gums and novel ingredients [38], many gluten-free industrial products available on the market exhibit a low nutritional quality, poor mouth feel or flavor [39] and, no less important, are particularly expensive. As a consequence, the research interest to develop gluten-free products has significantly increased in recent years. The pilot study by Rotsch [40] showed that bread could be prepared from starch and gel-forming substances, since starch, combined with water, at a temperature included between 60 and 80 °C, increases in volume, in a sort of swelling (a process called gelatinization) [41,42,43]. This process increases the product consistency by mimicking the gluten viscoelastic properties. Thickeners and gums/hydrocolloids, derived from various seeds, fruits, or plant extracts, could be also added in these formulations to improve water retention, texture and appearance properties [44,45,46,47]. Among different types of flours, present on the market and rich in starch, rice and corn flour are the best suited to the gelatinization process. These starches, are polysaccharides composed of amylose and amylopectin, present in the original product in the percentages equal to 20%–30% and 70%–80%, respectively.

The amylose is a linear polymer of glucose in which the glucose units are held together by α (1→4) bonds, whilst the amylopectin is a branched polymer of glucose, similar to amylose, structured with side chains that are grafted every 24–30 glucose units through α (1→6) bonds. Amylopectin is the most responsible for the gelatinization of starch granules in the presence of water and heat. During this process a fraction of both amylopectin and amylose goes into solution, however, the starch gelatinization is inversely proportional to the amylose content [48,49]. From a nutritional perspective, the more starch is gelatinized (because of its reduced amount of amylose), the more it is hydrolysable by alpha-amylase, with an increase of the glycemic index [50,51]. In contrast, with a greater amylose content, the gelatinization process is reduced, as well as the glycemic index. Gluten-free food prepared with corn and rice starch have a high glycemic index [52,53] and increase the risk to develop metabolic syndromes in celiac patients [54,55]. In fact, it is well known from epidemiological studies that the daily consumption of high glycemic index food is correlated with the risk to develop cardiovascular disease, obesity and diabetes [56,57,58,59,60]. Furthermore, the use of palm oil, cream powder, microencapsulated high-fat powder, and low-fat dairy powders [39,40], aimed to improve the palatability of cereal-based gluten-free products, renders these products highly caloric.

## 5. Recent Advances in Formulation of Cereal-Based Gluten-Free Food

In addition to the high glycemic index and caloric density of cereal-based gluten-free food, these products generally are not enriched/fortified, so that, they may not contain the same levels of nutrients, as the natural wheat grains. Therefore, uncertainty still exists whether celiac patients compliant with gluten-free therapy have a nutritionally balanced diet. Grehn *et al*. screened the intake of nutrients and foods of 49 adults diagnosed with coeliac disease and following a strict gluten-free diet [61]. They had a lower intake of fibre when compared to a control group on a normal diet. In their studies with coeliac adolescents, Mariani *et al*. [62] concluded that adherence to a gluten-free diet worsens the nutritionally unbalanced diet in adolescents, as it has been thereafter confirmed by Thompson *et al*. [63]. For these reasons several studies investigated the preparation of a new generation of cereal-based gluten-free food. The enrichment of baked products with dietary fibres and devoid of gluten has been the goal of various technologist teams. Gallagher *et al*. incorporated inulin (8% inclusion level) into a wheat starch-based gluten-free formulation [64]. Inulin is a non-digestible polysaccharide that is classified as a dietary fibre. It also acts as a prebiotic by stimulating the growth of “healthy” bacteria in the colon [65]. When added to wheat bread, inulin improves loaf volume, increases dough stability and produces a uniform, and finely grained, crumb texture [66].

Gambus *et al*. replaced corn starch with amaranth flour to enhance the protein and fibre contents of gluten-free breads [67]. At a 10% replacement level, protein and fibre levels increased by 32% and 152% respectively, whilst sensory quality was unaffected. Taylor and Parker discussed the use of quinoa in the production of enriched gluten-free bakery goods [68]. Both quinoa and amaranth are *dicotyledonous* species that are not related to the actual cereals (*monocotyledons*), such as wheat, barley and rice. They are also called pseudocereals, since these species produce small seeds that resemble those of cereals. The grain of pseudocereals does not contain gluten proteins but it is rich in proteins with high biological value (albumins and globulins) and contains carbohydrates that can be considered nutraceuticals, as they have cholesterol- [69,70] and glycemic-lowering effects, and induce a reduction of free fatty acids [52]. Importantly, the total absence of immune toxicity of amaranth storage protein for celiacs patients has been demonstrated [71], and several studies were carried out in order to improve the structural properties of quinoa and amaranth as ingredients for bread, pasta and crackers [72,73,74].

## 6. Nutritional Quality of Wheat Flour: Advantages in Introducing a Cereal-Based Gluten-Free Food in Diet of Celiacs

The therapy with gluten-free products, besides the risk of nutrient deficiency and metabolic syndrome, as described above, entails the difficulty of maintaining the cure over time. Reduced palatability and taste of gluten-free food create enormous limitations in the diet of patients. To solve these issues, numerous studies are currently devoted to the use of *in vitro* detoxified flour or flour from ancient wheat cultivars, in the formulation of pasta and baked goods.

### 6.1. Nutritional and Health Properties of Hexaploid and Tetraploid Wheat

Worldwide, the number of people who eat wheat for a substantial part of their diet reaches several billions. Because of the high content of starch, (about 60%–70% of the whole grain and 65%–75% of white flour) wheat is often considered no more than a source of calories. Despite its relatively low protein content (usually 8%–15%), wheat still provides as much protein for human and livestock nutrition as the total soybean crop (as calculated in reference [75]. However, the lysine content of wheat is low and varies significantly from grain to flour [15]. Grain of high protein content has very low content of lysine approximately 30 mg g^−1^ protein [76]. Wheat is a source of minerals such as Zn (20–30 mg Kg^−1^) and Fe (30–36 mg Kg^−1^), contributing to 44% of the daily intake of iron (15% in bread), and 25% of the daily intake of zinc (11% in bread) in the UK [77] Wheat is also a source of selenium which varies widely from about 10 µg Kg^−1^ to over 2000 µg Kg^−1^ (FAO/WHO, 2001; [78]). The concentration of selenium in wheat is largely determined by the availability of this element in the soil. Wheat produced in Western Europe may contain only one-tenth of the selenium that is present in wheat grown in North America. Wheat also contains a range of components with established health benefits that are concentrated, or solely located, in the bran. In addition, the following components are either present in low amounts, or completely absent, and with a large variation in their concentrations:

- **lignans**, a group of polyphenols with phytoestrogen-like activity, present at levels up to 10 µg g^−1^ in wholemeal wheat and almost 20 µg g^−1^ in the bran [79];
- **phenolic acids** that in wholemeal range up to almost 1200 µg g^−1^ [80]. They represent, quantitatively, the major group of phytochemicals in the wheat grain and are derivatives of either hydroxibenzoic acid or hydroxycinnamic acid. Epidemiological studies indicate that phenolic acids have a number of health benefits which may relate to their antioxidant activity; furthermore, a high correlation between the total antioxidant activities of grain and their phenolic acid contents has been reported [81,82];
- **folates** that in wholemeal varied from 364 to 774 ng g^−1^ dry weight in winter wheats and from 323 to 741 ng g^−1^ dry weight in spring wheats, and positively correlated with the bran yield [83];
- **dietary fibre** derives from polymers of wheat endosperm cell wall: they are constituted mainly by arabinoxylans (approximately 70%) and (1-3) (1-4)β-_D_-glucans (approximately 20%). The arabinoxylans are present in both soluble and insoluble forms, being the former considered to have health benefits [84,85]. However, insoluble fibre may also favour the delivering phenolic antioxidants into the colon, with a reduction of colon-rectal cancer risk [86]. Gebruers *et al*. showed wide variation in the contents of total and water-extractable arabinoxylans in both white flour and bran fractions [87]. Similarly Ordaz-Ortiz *et al*. showed variation from 0.26% to 0.75% dry weight in the content of water-extractable arabinoxylan in 20 French wheat cultivars and from 1.66% to 2.87% dry weight in total arabinoxylans [88].

### 6.2. Ancient Wheats

*Triticum monococcum*, was the first wheat to be cultivated by man and is a diploid species characterized by the presence of the AA genome. Due to the simplicity of its genome, *Triticum monococcum* has attracted the interest of the scientific community on nutritional and health aspects in relation to celiac disease. If we consider, in fact, that for each genome (AA, BB and DD) there are dozens of genes coding for prolamins in wheat caryopses, it is evident that in *Triticum monococcum* the mere presence of the AA genome encodes for a reduced variety of gluten proteins (and of potential immune toxic peptides). Recent evidence shows that prolamins of some *Triticum monococcum* cause a reduced inflammatory effect in celiac patients [89,90,91,92], particularly for the inability to activate the innate branch of immune cells [89], or to induce apoptosis of enterocytes [93]. The reduced, or absent, toxicity of *Triticum monococcum* prolamins can then be interpreted in two ways, namely via (*i*) a low presence of toxic peptides; (*ii*) an abundant presence of protective sequences. However, other *in vitro* and *in vivo* studies have warned about the safety of some monococcum cultivars for celiac patients [94,95], thus suggesting that the immune toxicity for celiacs might strictly depend on the specific *Triticum monococcum* varieties.

The search of naturally detoxified, or less toxic, ancient grains is of great interest for their potential use in the general diet to prevent disease in those individuals at high risk to develop gluten intolerance.

Regarding the technological properties, though some studies have considered *Triticum monococcum* not suitable for bread and pasta production [96,97], it has been demonstrated that the aptitude to bread-making is extremely different and depends on the specific varieties analyzed [98,99]. Studies carried out at an industrial level have shown that pasta made with 100% of *Triticum monococcum* flour has very low loss of starch during cooking comparable to that made from durum wheat semolina [23]. These findings on the bread- and pasta-making aptitude of *Triticum monococcum* flours are encouraging, and indicate a real chance to select for ancient varieties with superior technological characteristics and reduced toxicity for celiacs.

### 6.3. Detoxification of Wheat Gluten

Numerous studies are currently devoted to prepare pasta and baked goods made from wheat flours modified in order to eliminate, or reduce, the immune toxicity of gluten proteins (detoxification process).

A first method, using endopeptidase of bacterial origin during the preparation of wheat flour dough, results in the complete degradation of gluten peptides including those that are strongly immune toxic for celiacs [100]. Such an approach, carrying out a total destruction of the gluten network, reduces the technological properties (viscoelasticity) of dough and, consequently, of pasta or baked goods, unless the flour is integrated with structuring agents, as pre-gelatinized starch, emulsifiers or hydrocolloids.

Another method to detoxify gluten proteins uses the specific transdamidation of toxic epitopes done by the tissue-transglutaminase of microbial origin (*Streptomyces mobaraensis)* in the presence of lysine methyl ester [101]. This method has the great advantage of blocking the immunogenicity of T cell epitopes (as demonstrated in an *in vitro* assays using intestinal T cell from celiacs), and more importantly, it keeps intact the gluten network and preserves the technological properties of the flour. Furthermore, this procedure uses an enzyme largely employed in the food industry for improving the texture of foods. A preliminary 90-days trial made with CD subjects in remission consuming bread slices with transamidated gluten indicated that only a subgroup of celiacs exhibited clinical symptoms compared to subjects consuming the toxic gluten [102]. The researchers have now implemented the transamidation reaction in order to reach protection in the great majority of CD volunteers that eat detoxified wheat flour.

## 7. Conclusions

The gluten proteins contained in wheat flour are crucial during the bread and pasta making process, since they confers to the dough its viscosity and elasticity. In addition, wheat flour provides to foodstuffs important nutritional components, including dietary fibre, vitamins, and minerals. These properties account for wheat being cultivated by man in such enormous quantities throughout the world. However, gluten proteins are also responsible for a very common, and in most cases very severe, intolerance in a large number of individuals. To date the only safe and efficacious therapy for people with celiac disease is the long-life avoidance of gluten from the diet.

The replacement of the unique technological properties of wheat gluten represents the major task of industry for providing high quality gluten-free foods, such as pasta, bread and baked products, in terms of structure, loss of starch during cooking, and optimal cooking time.

Nutritional studies on people with CD on a gluten-free diet revealed several nutrient deficiencies, particularly of vitamins and minerals, as well as an increased of obesity risk, this latter due to the high glycemic index of the gluten-free diet. However, despite the numerous studies, it has not yet been possible to offer celiac patients an alternative diet therapy, based on high nutritional and tasty cereals that are naturally gluten-free. Foodstuffs made with technologically detoxified wheat flour, or with wheat varieties with naturally low content of toxic gluten sequences, or more suitable to gastrointestinal enzymatic degradation, would be a much-desired dietary therapeutic alternative. An extensive production of foodstuffs made with detoxified wheat flour that could be commonly consumed not only by those people suffering gluten intolerance, but also by the rest of the population could be envisaged; the large diffusion of such products could have the goal, in a totally innovative way, of reducing the immune sensitization to gluten and, likely, decreasing the incidence of celiac disease.
